# Infrared thermography: A potential noninvasive tool to monitor udder health status in dairy cows

**DOI:** 10.14202/vetworld.2016.1075-1081

**Published:** 2016-10-15

**Authors:** M. Sathiyabarathi, S. Jeyakumar, A. Manimaran, G. Jayaprakash, Heartwin A. Pushpadass, M. Sivaram, K. P. Ramesha, D. N. Das, Mukund A. Kataktalware, M. Arul Prakash, R. Dhinesh Kumar

**Affiliations:** 1 Department of Livestock Production and Management, Livestock Research Centre, Southern Regional Station, ICAR - National Dairy Research Institute, Bengaluru - 560 030, Karnataka, India; 2 Department of Animal Nutrition, College of Veterinary and Animal Sciences University, Mannuthy - 680 651, Kerala, India; 3 Dairy Engineering Section, Southern Regional Station, ICAR - National Dairy Research Institute, Bengaluru - 560 030, Karnataka, India; 4 Dairy Economics and Statistics Section, Southern Regional Station, ICAR - National Dairy Research Institute, Bengaluru - 560 030, Karnataka, India; 5 Dairy Production Section, Southern Regional Station, ICAR - National Dairy Research Institute, Bengaluru - 560 030, Karnataka, India; 6 Department of livestock Production and Management, University Research Farm, Tamil Nadu Veterinary and Animal Sciences University, Chennai - 600 051, Tamil Nadu, India

**Keywords:** cows, infrared thermography, mastitis diagnosis, udder health, udder surface temperature

## Abstract

The animal husbandry and livestock sectors play a major role in the rural economy, especially for the small and marginal farmers. India has the largest livestock population in the world and ranks first in the milk production. Mastitis is the most common and expensive infectious disease in dairy cattle. The global economic losses per year due to mastitis amounts to USD 35 billion and for Indian dairy industry ₹6000 crores per year. Early detection of mastitis is very important to reduce the economic loss to the dairy farmers and dairy industry. Automated methods for early and reliable detection of mastitis are currently in focus under precision dairying. Skin surface temperature is an important indicator for the diagnosis of cow’s illnesses and for the estimation of their physiological status. Infrared thermography (IRT) is a simple, effective, on-site, and noninvasive method that detects surface heat, which is emitted as infrared radiation and generates pictorial images without causing radiation exposure. In human and bovine medicine, IRT is used as a diagnostic tool for assessment of normal and physiological status.

## Introduction

India ranks first in the world total milk production, the total milk production in the country being 146.3 million tons in 2014-2015 [1]. Mastitis ranks first among the diseases of dairy cows with high prevalence and incidence rate, which causes severe economic losses to the dairy farmers. Mastitis is the inflammation of udder tissue causing pathological changes in udder parenchyma and characterized by physical, chemical, and microbiological changes in milk. The losses are either due to loss of milk production (temporary or permanent), poor milk quality, discarding of milk from affected animals, and reduced milk productive life or culling of the cow. Delay in the detection of subclinical mastitis and lack of appropriate and accurate technique are contributing to the higher incidence of clinical mastitis. The loss due to subclinical mastitis is higher than the clinical mastitis and milk yield loss due to mastitis ranges from 100 to 500 kg/cow per lactation [2].

Several diagnostic tests exist for detection of mastitis, *viz*., milk color, pH test, Electrical Conductivity (EC), California Mastitis Test (CMT), Somatic Cell Count (SCC), culture test, biomarkers, proteomic technique, and immunoassay. Biosensor system that analyzes lactose and EC has been claimed to have a sensitivity of >90% for identifying quarters with ≥100,000 cells/mL [3]. These techniques are subjective, laborious, and not adequately precise for detection of early signs of the disease. Automated methods for early and reliable detection of mastitis are currently in focus. Infrared thermography (IRT) is a simple, effective, on-site, and noninvasive method that detects surface heat, which is emitted as infrared radiation and generates pictorial images without causing radiation exposure. In bovine medicine, IRT is used for early detection of subclinical mastitis [4] (Table-1), heat detection and prediction of ovulation in cows [5,6], detection and assessment of lameness [7,8], assessment of animal welfare, and feed utilization efficiency [9]. This review presents a more comprehensive understanding of the potential application of the IRT technique to monitor udder health status and early detection of mastitis in dairy animals.

**Table-1:** IRT camera models used in various studies to assess udder health status in dairy cattle.

Name of the IRT camera	Application	References
FLIR Inframetrics 760	Used to measure daily variation in the UST of dairy cows in relation to mastitis	Berry *et al.* [43]
ThermaCam545	Used to identify the changes of teat temperature in machine milking	Vegricht *et al.* [50]
ThermaCam P25	Used for early detection of mastitis in dairy cows	Colak *et al.* [16]
IRI 4010	*E. coli* endotoxin intramammary infusion in late lactation and changes in the UST	Costa *et al.* [51]
FLIR 760 IR scanner	*E. coli* infusion and UST changes	Metzner *et al.* [45]

*E. coli=Escherichia coli*.

## Udder Health and its Importance

Mastitis is categorized into contagious mastitis and environmental mastitis. Contagious mastitis is caused by bacteria, and it can be divided into clinical mastitis, subclinical mastitis, and chronic mastitis. Clinical mastitis (peracute mastitis, acute mastitis, and subacute mastitis) is characterized by the presence of gross inflammatory signs (redness, heat, swelling, pain, and loss of function). The subclinical mastitis is characterized by the change in milk composition with no signs of gross inflammation or milk abnormalities. In chronic mastitis, inflammatory process, that exists for months and may continue from one lactation to another. Environmental mastitis is caused by organisms such as *Escherichia coli* [10]. Mastitis in dairy animals leads to economic losses to the dairy farmers and to the dairy industry as a whole in different forms, *viz*., reduction in milk production (70%), premature culling (14%), veterinary expenses (9%), and milk discarded or low graded (7%). The global estimated economic losses per year due to mastitis amounts to USD 35 billion and INR.6000 crores for the Indian Dairy Industry [11].

## Diagnosis of Mastitis: Traditional versus Recent Trends

**Figure-1:**
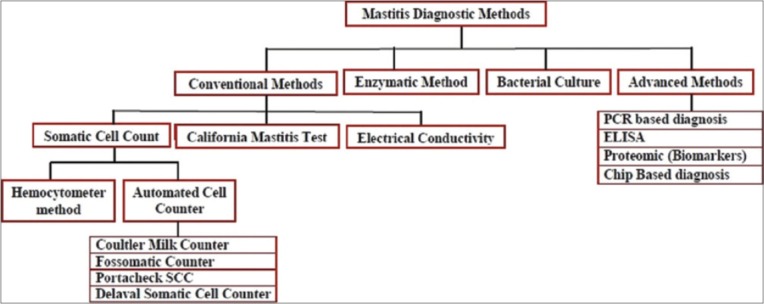
Traditional versus recent trends in mastitis diagnosis of dairy cattle.

Among various indicators of mastitis, *viz*., gross examination of udder, milk color, pH test, EC, CMT, SCC, culture test, biomarkers, proteomic technique, and immunoassay (Figure-1). SCC in milk is highly accepted as an important and rapid indicator of the inflammatory status of the udder. Somatic cells are milk-secreting epithelial cells that shed from the lining of the gland, and white blood cells that have entered the mammary gland in response to injury or infection. The milk somatic cells include 75% leukocytes and 25% epithelial cells. Mammary gland infection level (mastitis), stage of lactation, age, breed, parity, season, stress, diurnal variations, milk transportation, and management are some factors that influence milk SCC at individual and herd level. The SCC is lower than 1×10^5^ cells/mL of milk from a healthy mammary gland, whereas bacterial infection can cause it to increase to above 1×10^6^ cells/mL [12]. Mean normal values of SCC in the milk of indigenous, *viz*., Deoni 1.95±0.24 lakhs/mL, Ongole 1.57±0.22 lakhs/mL, and HF crossbred 4.14±0.17 lakhs/mL, dairy cows [13].

### IRT

**Figure-2:**
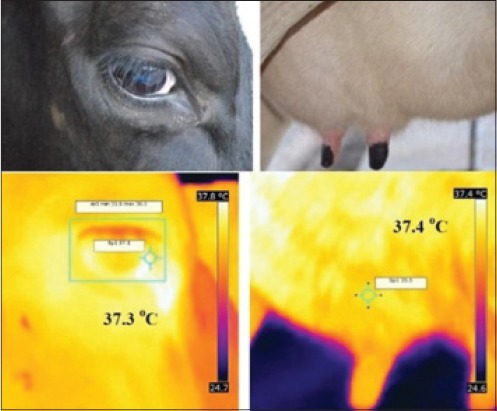
Infrared thermogram and visual image of eye and udder surface.

**Figure-3:**
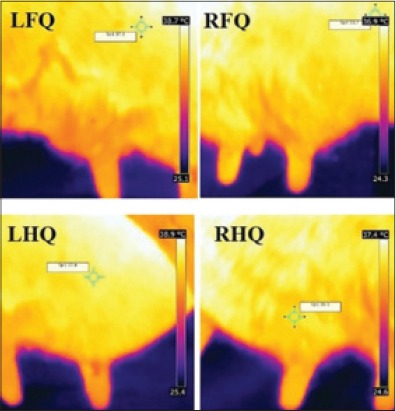
Infrared thermogram of udder quarters from the lateral side. LFQ: Left front quarter, RFQ: Right front quarter, LHQ: Left hind quarter, RHQ: Right hind quarter.

Infrared was discovered by a British astronomer, Sir William Herschel, in 1800. Infrared is an electromagnetic wave, with wavelength ranging from 700 nm to 1 mm. Every object, whose surface temperature is above absolute zero, radiates energy at a wavelength corresponding to its surface temperature. All objects emit infrared radiation proportional to their body temperature according to Stefan-Boltzmann [4]. The process in which energy is emitted as waves is known as radiation. Emissivity refers to an object’s ability to emit radiation [14]. The total radiation energy emitted or absorbed by the animal body depends on the emissivity of the skin, the emissivity of most of the objects is <1 [4]. Early infrared imaging systems were developed during the 1940s and found use in industry and medicine in 1959 [15]. IRT is a simple, effective, on-site, and noninvasive method that detects surface heat, which is emitted as infrared radiation and generates a pictorial image without causing radiation exposure. In a thermogram, the warmest region appears as white or red, whereas the coolest region appears as blue or black (Figures-2 and 3) [16].

## Application of IRT

Thermal imaging has got a potential application in industry, agriculture, and medicine. IRT is employed for assessment of structures, locating the source of distress, assessment of damage potential in concrete and building materials structures, sensing moisture ingress and flow through pipes, etc. In agriculture, IRT is employed for assessing seed viability, estimating soil, water status, estimating crop water stress, scheduling irrigation, determining disease and pathogen of affected plants, estimating fruit yield and evaluating the maturity of fruits and vegetables, etc. There are several applications of IRT in the field of human medicine such as assessment of neurological disorders, open-heart surgery, vascular diseases, reflex sympathetic dystrophy syndrome, urology problems, mass fever screening, arthritis, and evaluation of breast cancer [14].

## Application of IRT in Veterinary Medicine

In bovine medicine, IRT is used for diagnostic purposes, assessment of animal welfare, and feed utilization efficiency. IRT is widely used to identify localized areas of inflammation, such as mastitis in lactating cows [17-19], foot and mouth disease [20], assessment of tissue damage and healing due to hot versus cold branding in cattle [21], Laminitis [7,8], detection of bovine viral diarrhea in calves [22], monitoring respiratory disorders [23], *Actinobacillus pleuropneumoniae* infection in pigs [24], detection of estrus and prediction of ovulation in cattle and gilts [5,6,25,26], to assess the effect of scrotal temperature on sperm production in bulls [27-29], assessing meat quality in pigs [30], for identification of stress [31], measurement of feather cover [32], effects of machine milking on teat and udder [33], surface temperature, estimation of heat and methane production in dairy cattle [34], screening of cattle for feed utilization efficiency [9,35], pregnancy diagnosis in mare and wild animals [36,37], evaluation of thermal status of neonatal pigs [38], monitoring stress during animal transit and welfare in wild animals [39], assessment of surface temperature of buffaloe bulls and its correlation with rectal temperature [40] and for the evaluation of thermoregulatory capacity of dairy buffaloes [41].

## Application of IRT to Assess Pathophysiological Status of Udder in Dairy Cows

### Milking process

Aljumaah *et al*. [42] investigated the effect of machine milking on normal physiological parameters of lactating camels and concluded machine milking had no effect on average rectal (37.88±0.23°C) and vaginal temperatures (37.94±0.14°C), as well as respiratory (16.12±0.23 breath/min) and heart rates (56.78±1.89 beat/min). A significant decrease in udder (−1.0°C) and teat (−1.6°C) surface temperatures was detected 1 h immediately after milking as a consequence of machine milking. Similarly, Alejandro *et al*. [33] studied the effect of machine milking on teat tissue changes in Murciano-Granadina goats, and they found that machine milking caused a significant increase (p<0.05) of the mean temperature by 6.6, 4.9, 2.5, and 1.5°C at the tip, 1, 2, and 3 cm from the teat end.

Poikalainen *et al*. [4] determined the possibilities of cow’s thermal profile registration at free stall housing, investigated the possibilities of automatic cow’s udder thermograms registration at milking parlor and milking robot, and to compare the temperatures of the udder before and after milking. The authors found no significant difference between temperature of left and right udder quarters before and after milking, and they concluded that udder surface temperature does not depend on milking.

### Subclinical and clinical mastitis

Polat *et al*. [19] determined the interrelationships among mastitis indicators and evaluated the mastitis detection ability of IRT in comparison with the CMT in Brown Swiss dairy cows. Subclinical mastitis quarters showed 2.35°C greater skin surface temperature than healthy quarters (Table-2). The UST was positively correlated with CMT score and SCC. There was an exponential increase in SCC and a linear increase in UST as the CMT score increased. The authors concluded that IRT can be employed as a noninvasive, quick tool, for screening subclinical mastitis via measuring UST, with a high predictive diagnostic ability similar to CMT when microbial culturing is unavailable. Berry *et al*. [43] investigated magnitude and pattern of daily variation in the UST of Holstein-Friesian dairy cows in various stages of lactation using IRT technology. Measures of SCC were below 250,000 with the exception of one animal; it had an average SCC below 250,000 before the trial; however, during the trial, the temperature and SCC rose to levels indicative of subclinical mastitis. No visible signs of mastitis were evident in its foremilk, and bacteriological analysis did not detect any pathogenic organisms. Porcionato *et al*. [44] used IRT for the detection of subclinical mastitis in Gir cows of the second and third lactation. The researchers using IRT measured the surface temperature of teat three heights (upper, median, and lower) and correlated with milk SCC microbiological tests for pathogen. There was the difference in temperature between the heights, with higher temperature values in the upper region than in the other regions of the teats (median and lower). There was no significant correlation between log SCC and UST measured or between the type of pathogens and UST in three different heights. The authors concluded that the use of thermal camera allowed the identification of variations in skin surface temperature in different heights of the udder of Gir cows. However, this technique was not effective in the diagnosis of subclinical mastitis. Martins *et al*. [35] evaluated the use of IRT for mastitis diagnosis in relation with SCC and milk composition in sheep. The UST was higher for subclinical mastitis group. The clinical mastitis group had highest fat and protein levels as well as the lowest lactose level. The authors concluded that infrared udder temperatures can be a good auxiliary diagnostic method to mastitis in sheep, principally to subclinical mastitis. Therefore, thermography is a promissory technique for subclinical mastitis diagnosis in sheep.

**Table-2:** Body (i.e., eye) and udder surface temperature differentials of dairy cows affected by clinical and subclinical mastitis.

Mastitis condition	Difference between body and UST	References
Clinical mastitis	1-1.5°C	Hovinene *et al.* [18]
Subclinical mastitis	2.35°C	Polat *et al.* [19]

Hovinen *et al*. [18] tested thermal camera for its capacity to detect clinical mastitis, by experimentally inducing mastitis in cows with 10 µg of *E. coli* lipopolysaccharide. The thermal camera was successful in detecting 1-1.5°C temperature change on udder skin associated with clinical mastitis in all cows because the temperature of the udder skin of the experimental and control quarters increased in line with the rectal temperature (Table-2). Colak *et al*. [16] conducted an experiment to determine the merit of IRT for early detection of subclinical mastitis. As the CMT score increased, quarters skin surface temperature increased linearly. IRT was sensitive enough to perceive changes in skin surface temperature in response to the severity of the mammary gland infection as reflected by the CMT score. The study suggests that IRT can be employed for screening dairy cows for mastitis. Willits [17] suggested that IRT is a suitable tool for screening and early detection of mastitis in dairy herds. Metzner *et al*. [45] compared different algorithms for the evaluation of udder skin thermogram for detection of *E. coli*-induced acute mastitis in dairy animals. Analysis of udder surface temperature using different geometric analysis tools (polygons, rectangles, and lines) and descriptive parameters (minimum, maximum, range, arithmetic mean, and standard deviation) revealed that significant changes can be detected best through using the analysis tool “polygons” and the descriptive parameter “maximum.” They also suggested that IRT was only valid for testing of the hind quarters as this combination yielded the highest correlation with rectal temperature. The greatest difference in the temperatures between control and *E. coli* inoculated quarters (2.06°C) was found in “polygons” and “rectangles” using “maximum.”

## Factors Influencing the IRT Imaging of Udder

Before taking IRT image, animal must be tied properly in standing position under shaded shed. Udders are then brushed or wiped with the clean towel to remove dung and dirt. Mechanical brushing causes the changes in udder skin surface temperature [43]. After brushing or wiping, the udder quarter is allowed resting for 10-15 min before images are taken. Images are taken in the standing position at a distance of 0.6-1 m [46,47]. Images of the front quarters are taken from the lateral side of the animal, and the hind quarters were taken from the lateral or posterior side. IRT images must be taken out of direct solar radiation (sunlight) and wind speed. Increased solar radiation and wind speed cause the rise in the UST [48]. Animal factors such as parity, stage of lactation, and pregnancy may also influence the IRT udder surface temperature pattern. Berry *et al*. [43] suggested that IRT was only valid for monitoring hind quarters. Front quarters possibly display different patterns of surface temperature, especially since the surface temperature might be more or less affected by thermal radiation emanating from the medial aspect of the adjacent legs. Berry *et al*. [43] reported that the hind quarters were more exposed to environmental temperature than the front quarters. Similarly, Chun-He *et al*. [49] found the normal temperature distribution between rear left and rear right quarters. However, there was no significant difference in UST among the four quarters [19].

## Future Areas of Research

Application of IRT technique would require a great deal of basic data for different breeds under a wide variety of climatic and environmental conditions and management system. The analyzed IRT data would help to establish breed and location specific normal thermographic profile. Detailed studies are needed to observe the influence or interaction of interquartile difference, breed difference, period of lactation/parity, milk yield, stage of lactation, day-to-day variation of the UST, within day variation of the UST, body temperature versus UST, clinical condition (mastitis – subclinical/clinical) and reproductive status (pregnant/nonpregnant), causative organism, and their virulence factors resulting in different forms of mastitis.

## Conclusion

IRT is a noninvasive, handheld tool with specific analyzing software, has remarkable priorities in precision dairy farming and veterinary medicine (diagnostics of mastitis, leg injuries, body surface damages, milking hygiene, etc.). IRT could prove conclusively as an important diagnostic tool in addition to conventional techniques available for monitoring udder health and early detection of mastitis.

## Authors’ Contributions

SJ, AM, HAP, MS, KPR, DND, and MAK hypothesized the concept of this review paper. MS, SJ, and AM prepared the manuscript. GJ, MAP, and DKR assisted in collecting and compiling the resource material and in manuscript preparation. All authors read and approved the final manuscript.
